# Household- and community-level factors of zero vegetable or fruit consumption among children aged 6–23 months in East Africa

**DOI:** 10.3389/fnut.2024.1363061

**Published:** 2024-06-19

**Authors:** Abel Endawkie, Alemu Gedefie, Amare Muche, Anissa Mohammed, Aznamariam Ayres, Dagnachew Melak, Eyob Tilahun Abeje, Fekade Demeke Bayou, Fekadeselassie Belege Getaneh, Lakew Asmare

**Affiliations:** ^1^Department of Epidemiology and Biostatistics, School of Public Health, College of Medicine and Health Sciences, Wollo University, Dessie, Ethiopia; ^2^Department of Medical Laboratory Science, College of Medicine and Health Sciences, Wollo University, Dessie, Ethiopia; ^3^Department of Pediatrics and Child Health Nursing, College of Medicine and Health Sciences, Wollo University, Dessie, Ethiopia

**Keywords:** household and community level factors, zero vegetable or fruit consumption, children, East Africa, multilevel analysis

## Abstract

**Introduction:**

The World Health Organization recommends that children aged 6–23 months should consume a diversified diet, including fruits and vegetables, during each meal. However, low consumption of fruits and vegetables contributes to 2.8% of child deaths globally. The literature review indicates limited research on factors that affect zero vegetable or fruit consumption among children aged 6–23 months in East Africa. Therefore, this study aimed to investigate the household- and community-level factors determining zero vegetable or fruit consumption among children aged 6–23 months in East Africa.

**Method:**

The study analyzed cross-sectional secondary data from the recent rounds of demographic and health surveys conducted in East Africa from 2015 to 2023. The weighted sample comprised 113,279 children aged 6–23 months. A multilevel mixed-effect analysis was used, measuring the random variation between the clusters based on the intra-cluster correction coefficient, median odds ratio, and proportional change variance. Adjusted odds ratio with a 95% confidence interval was reported while considering variables having a *p* < 0.05 as statistically significant.

**Results:**

The overall prevalence of zero vegetable or fruit consumption among children aged 6–23 months in East Africa was 52.3%, with Ethiopia showing the highest prevalence (85.9%). The factors associated with zero vegetable or fruit consumption were maternal educational level, number of household members, short birth interval, multiple births, sex of the household head, household wealth index, community-level maternal literacy, community-level wealth index, and countries.

**Conclusion:**

Considering the high overall prevalence of zero vegetable or fruit consumption among children aged 6–23 months in East Africa, overlooking this nutritional gap among children is a serious oversight. Therefore, efforts should be geared toward improving individual- and community-level maternal literacy. In particular, nutrition and public health organizations should support low-income communities to achieve vegetable or fruit consumption for infants and young children.

## Introduction

In 2021, the WHO and the United Nations Children's Fund (UNICEF) defined zero vegetable or fruit (ZVF) consumption among children aged 6–23 months as a situation where a child did not consume any vegetables or fruits on the previous day (1).

The consumption of fruits and vegetables is crucial for the growth and development of children aged 6–23 months (2). Vegetables and fruits are rich in essential nutrients including vitamins, minerals, and dietary fiber, which contribute to the overall health and wellbeing of children (3). It is vital to introduce appropriate complementary feeding practices with diverse, nutrient-rich foods that include vegetables and fruits (4–6). Vegetables and fruits have consistently been emphasized in nutritional guidance because of their rich vitamin content for children aged 6–23 months (5, 7, 8). The inclusion of fruits and vegetables in the diet of infants is crucial in preventing stunting, establishing healthy dietary habits in adulthood, and providing long-term protection against non-communicable diseases (4, 5, 9, 10). Conversely, zero vegetable and fruit consumption can lead to short-term health problems (malnutrition) such as stunting, wasting, and being underweight. Furthermore, it can cause long-term health problems (chronic disease) such as cardiovascular diseases, diabetes, and certain types of cancer, including stomach cancer and colorectal cancer, in their later lives. Children who regularly consume vegetables and fruits are less likely to suffer from these health problems later in their life (3, 6, 11). However, despite these benefits, children's intake of vegetables often falls below the recommended levels globally (12). Approximately 1.7 million (2.8%) child deaths worldwide are attributable to low fruit and vegetable consumption (6). The global prevalence of ZVF consumption among children aged 6–23 months in 64 countries was 45.7%, with the highest proportion in Africa (12). A study in Sub-Saharan Africa (SSA) revealed that ZVF consumption among young children was 47%, with the highest prevalence in Ivory Coast (76%), Burkina Faso (75%), Chad (71%), Niger (71%), and Ethiopia (69%) (13). The WHO advises that children aged 6–23 months should have a diverse diet, including fruits and vegetables, during each meal (14).

Previous studies have found that children belonging to higher-income households (8, 13, 15) as well as those having mothers who are employed and more educated (8, 13) and those having mothers who have access to media (8, 9) were less likely to consume ZVF as compared to their counterpart.

Previous research on the consumption of vegetables and fruits was conducted using simple logistic regression, which may obscure cluster variation (4, 5, 7, 16–20). To address this limitation, using a multilevel analysis is crucial to identify cluster variation, considering the hierarchical nature of national demographic and health surveys. Our literature review indicates limited research that investigates the determinants of household- and community-level factors of ZVF consumption among children aged 6–23 months in East Africa. Such analyses can offer valuable insights for creating contextually relevant strategies and policies. Moreover, evidence from such research can contribute to achieving the sustainable development goal (SDG) target 2.2 aimed at ending all forms of malnutrition by 2030 (21). Therefore, this study aims to address the gap in the literature by investigating the household- and community-level factors determining ZVF consumption among children aged 6–23 months in East Africa. The study uses data from the recent rounds of demographic and health surveys (DHS) to provide empirical evidence on the subject.

## Methods

### Study design

This is a cross-sectional study based on secondary data obtained from the recent DHS conducted in East Africa.

### Study settings

Community-based cross-sectional surveys have been conducted between 2015 and 2023 among children aged 6–23 months in East Africa. In this study, we included data for Ethiopia, Tanzania, Rwanda, Uganda, Kenya, Comoros, and Burundi because the latest DHS data were available for these countries. Countries with no DHS data such as Somalia, Eritrea, South Sudan, Sudan, and Djibouti were excluded from the analysis. After authorization was granted via an online request explaining the purpose of our study, we obtained data for these countries from the DHS program's official database (https://dhsprogram.com).

### Data source

For this study, we extracted dependent and independent variables from the birth record (BR) dataset of the recent DHS data, which contains the full birth history of all women interviewed. The DHS is a nationally representative household survey conducted across low- and middle-income countries every 5 years. It comprises information on postnatal care, immunization, health, and nutrition, including breastfeeding, consumption of vegetables and fruits, and MDD data for children born in the last 5 years (22, 23).

### Source and study population

The source population included children aged 6–23 months 5 years before each survey in East Africa, whereas the study population comprised children aged 6–23 months in the selected Enumeration Areas (EAs).

#### Sample size and sampling method

The sample size was determined from the BR file in the DHS data of East African countries 5 years before the survey. The final sample size was 117,684 (weighted sample 113,279) children aged 6–23 months. DHS uses a two-stage stratified cluster sampling technique. In the first stage, a sample of EAs is selected independently from each stratum with proportional allocation stratified by residence (urban and rural). In the second stage, households are taken from the selected EAs using a systematic sampling technique.

#### Study variables

ZVF consumption with values 0 for “no” and 1 for “yes” was considered a dependent variable. If children aged 6–23 months consumed any vegetables or fruits on the previous day, ZVF consumption was categorized as “no,” whereas if they did not consume any vegetables or fruits on the previous day, based on the WHO and UNICEF 2021 guidelines, ZVF consumption was categorized as “yes” (1).

The following sociodemographic and economic-related factors were considered independent variables: individual-level factors such as maternal age, educational status, and marital status; household-level factors such as the number of household members, sex of the household head, age of the household head, and household wealth index; community-level factors such as place of residence, community-level wealth index, community-level maternal literacy, and countries of East Africa; and obstetric factors such as antenatal care (ANC) visits, birth interval, multiple births, and mode of delivery.

### Variable measurement

#### ZVF consumption

Children aged 6–23 months who did not consume any vegetables or fruits on the previous day are considered to have ZVF consumption based on the WHO and UNICEF 2021 guidelines (1).

#### Household wealth index

It is a composite measure of the cumulative living standard of a household using a combination of asset indicators, such as television, refrigerator, mobile telephone, availability of electricity, landline phone, bicycle, car, and cart, through principal components analysis (PCA) (24). In DHS data, the household wealth status is divided into five quintiles (i.e., poorest, poorer, middle, richer, and richest). In this study, we categorized the household wealth index into three levels: poor (poorest and poorer), average (middle), and rich (richer and richest).

#### Community-level factors

These factors are the physical and social environments surrounding individuals, households, or families that affect the probability of individuals engaging in specific behaviors. In this study, we examined community-level factors such as community-level wealth index, community-level maternal literacy, place of residence, and country.

#### Community-level wealth index

This index is calculated by summing the proportions of women belonging to households classified as the poorest and poorer wealth index categories and dividing it by the total household wealth index value of each cluster. If a household's wealth index value is equal to or greater than the mean, it is classified to be of a high poverty level. By contrast, if a household's wealth index value is less than the mean, it is categorized to be of a low poverty level. The mean is chosen as the cutoff point in this context because the poverty level at the community level follows a normal distribution, as indicated by the coefficient of skewness falling between −1 and 1.

#### Community-level maternal literacy

This measure is obtained by summing the proportions of mothers who have completed primary school and above levels and dividing it by the total maternal educational status value of each cluster. Mothers with an educational status equal to or above the mean are classified as having a high level of maternal literacy, whereas those with an educational status below the mean are categorized as having a low level of maternal literacy. The mean is chosen as the cutoff point in this context because the level of maternal literacy at the community level follows a normal distribution, as indicated by the coefficient of skewness falling between −1 and 1.

### Data processing and analysis

The data were extracted, cleaned, coded, and analyzed using the statistical software Stata version 17. The sample data were weighted before conducting further analysis. Descriptive statistics using frequencies, percentages, mean, and standard deviation was adopted to analyze the sociodemographic characteristics of the study participants, and the findings are presented using tables, figures, and narratives.

#### Multilevel mixed effect model

A multilevel analysis was conducted after verifying the data's eligibility for multilevel analysis by using the intra-cluster correction coefficient (ICC) = $\frac{\delta 2}{\delta 2+\;\pi 2/\;3}$, where δ^2^ indicates the estimated variance of clusters (25). The log of the probability of ZVF consumption was modeled using a two-level multilevel regression model as follows: Log[$-\frac{\pi ij}{1-\pi ij}-$] = β0+β1Xij+ B2 Zij+μj+ eij, where *i* and *j* are the household- and community-level units, respectively (26); X and Z refer to the household- and community-level variables, respectively; πij is the probability of ZVF consumption for the *i*th child aged 6–23 months in the *j*th household and community; and β's indicate the fixed coefficients, with β0 being the intercept. First, a bivariable multilevel logistic regression analysis was used, and variables with *p* < 0.2 were selected to develop six models as described below:

(1) Model-0 is an empty model or a null model
(2) Model-1 is a model for analyzing only individual-level variables
(3) Model-2 is a model for analyzing only household-level variables
(4) Model-3 is a model for analyzing only community-level variables
(5) Model-4 is a model for analyzing only household- and community-level variables
(6) Model-5 is a model for analyzing all individual-, household-, and community-level variables based on the cutoff points

The median odds ratio (MOR) in all six models (0–5) and the proportional change in variance (PCV) for five models (1–5) were used to measure the random effects and display the variation between the clusters. They are calculated as follows:

MOR = exp($\sqrt{2+\delta 2\;+\;0.6745}$) and PCV = $\frac{\text{δ}2\text{null model}-\text{δ}2\text{ of each model}}{\text{δ}2\text{null model}},$ where δ2 of the null model is used as a reference. Multicollinearity was checked among explanatory variables by using a standard error cutoff of ±2. No multicollinearity was confirmed as the standard errors were within ±2. The appropriateness of the mixed model was verified using model selection based on the Akaike information criterion (AIC) or Bayesian information criteria (BIC). Variables with a *p* < 0.05 in Model-5 were considered to be significantly associated with ZVF consumption.

### Ethical approval

No ethical approval was required because we used the demographic and health survey that de-identifies all data before making them public, and the DHS datasets are openly accessible. An authorization letter was sent to the Central Statistical Agency (CSA), and permission was obtained to download the DHS dataset from https://dhsprogram.com/. The dataset and all methods of this study were conducted according to the guidelines laid down in the Declaration of Helsinki and based on DHS research guidelines.

## Results

### Sociodemographic characteristics of mothers and children

A weighted sample of 113,279 children aged 6–23 months along with their mothers were included in the analysis. The mean age and standard division of the mothers were 34 and 5 years, respectively. Among these women, 35,398 (31.25%) were in the age group of 30–34 years and 31,727 (28.1%) could not read and write. Among the study participants, 105,612 (93%) were married (Table 1).

**Table 1:** Sociodemographic characteristics of the study respondents in East Africa using recent DHS data from 2015–2023 (weighted sample).

**Variable**	**Category**	**Frequency**	**Percent**
Maternal age category	15–19	7.3	0.01
	20–24	3, 354	2.96
	25–29	18, 962	16.74
	30–34	35, 398	31.25
	35–39	33, 333	29.43
	40–44	17, 715	15.64
	45–49	4, 509	3.98
Level of maternal education	No education	31, 727	28.01
	Primary	61, 806	54.56
	Secondary	13, 652	12.05
	Higher	6, 094	5.38
Marital status	Un-union	7, 667	7
	Union	105, 612	93
Number of household members	1–5	27, 647	24
	6–7	42, 335	37
	>8	43, 298	38.22
Number of children	0–4	112, 342	99
	>5	937	1
Sex of the household head	Male	90, 333	80
	Female	22, 947	20
Age of the household head	15–21 years	45	0.04
	22–34 years	29, 708	26.23
	35–64 years	79, 271	69.98
	>64 years	4, 255	3.76
Place of residence	Urban	20, 011	17.7
	Rural	93, 268	82.3
Household wealth index	Poor	50, 662	44.7
	Average	22, 783	20.11
	Rich	39, 834	35.2
Community-level wealth index	Low	51, 327	45.5
	High	61, 952	54.5
Community-level maternal literacy	Low	82, 285	72.64
	High	30, 994	27
Country	Burundi	16, 360	14.4
	Ethiopia	7, 168	6.3
	Kenya	26, 777	23
	Comoros	2, 875	2.5
	Rwanda	16, 969	15
	Tanzania	13, 199	12
	Uganda	29, 931	26

### Maternal and child health service utilization

The children's mean age and standard deviation were 11 and 4 months, respectively. Of the total number of children included in the study, 54,666 (48%) were female. Regarding the utilization of maternal health services among the study participants, 84,382 (97.4%) women had ANC visits related to delivery, and 103,956 (87%) delivered in private hospitals (Table 2).

**Table 2:** Characteristics of children and maternal health service utilization among the study respondents in East Africa using the recent DHS from 2015–2023 (weighted sample).

**Variable**	**Category**	**Frequency**	**Percent**
Age of the child	6–11 months	68, 807	61
	12–23 months	44, 472	39
Sex of the child	Male	58, 613	52
	Female	54, 666	48
Types of birth	Single	112, 236	99
	Multiple	1, 043	1
Birth interval	Less than 2 years	21, 719	19
	2 years	4, 724	4
	Greater than 2 years	868, 36	77
ANC visit	No	2, 280	2.6
	Yes	84, 382	97.4
Pregnancy type	Wanted	1, 098, 001	92
	Unwanted	9, 012	8
Modes of delivery	Vaginal	114, 117	91
	Cesarean section	11, 226	9
Place of delivery	Home delivery	21, 487	17
	Health facility	103, 956	83

ANC, Antenatal care.

### Prevalence of ZVF consumption in East Africa

**Figure 1:**
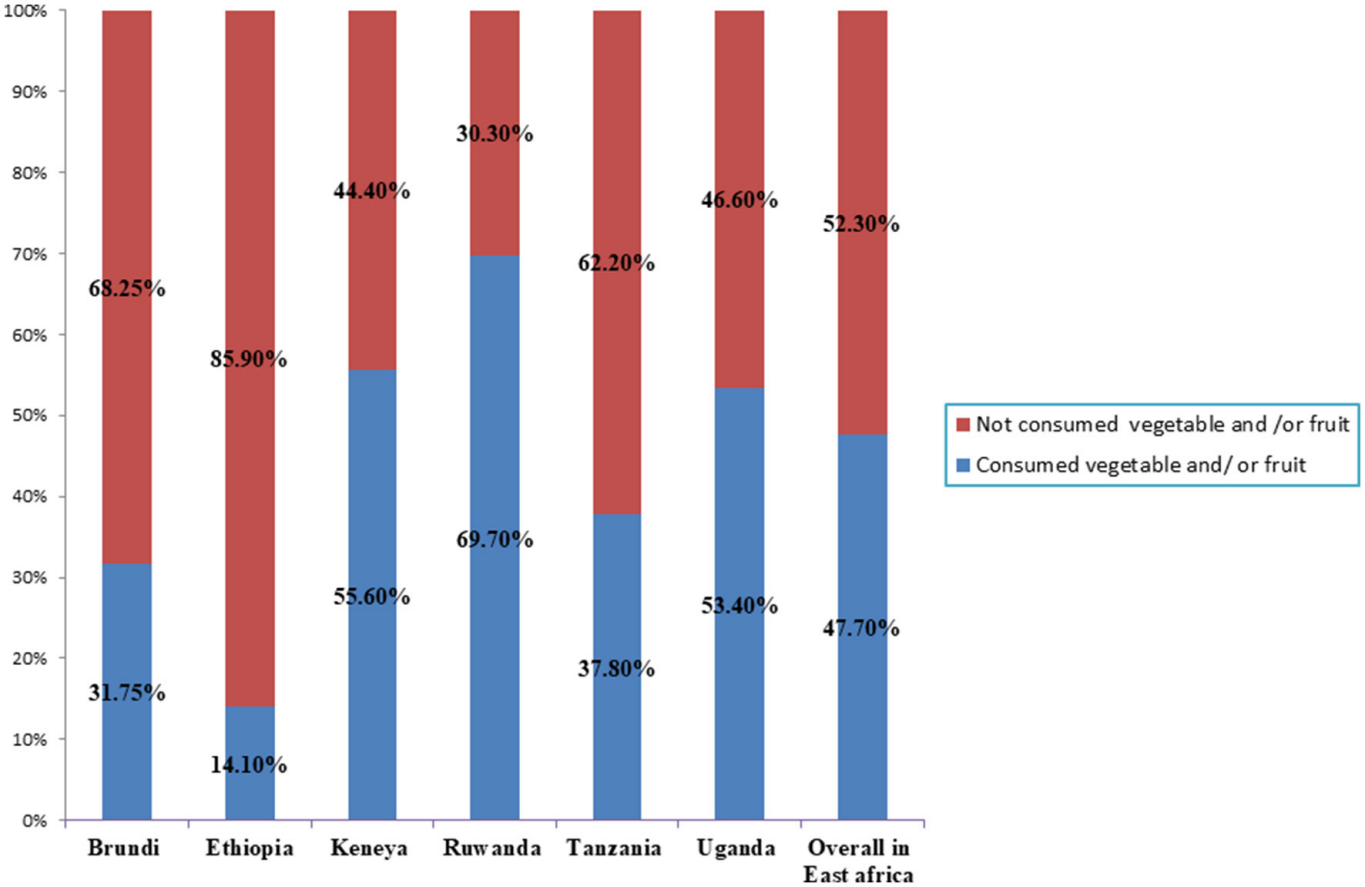
Zero vegetable and /or fruit consumption among children aged 6–23 month in east African countries using recent demographics and health survey.

The overall prevalence of ZVF consumption among 6–23 month-old children in East Africa was 52.3% (95% confidence interval (CI): 51.9%−52.6%). The prevalence of ZVF consumption among children aged 6–23 months was highest in Ethiopia (85.9%) and lowest in Rwanda (30.3%) (Figure 1).

### Distribution of ZVF consumption based on socioeconomic factors in East Africa

**Figure 2:**
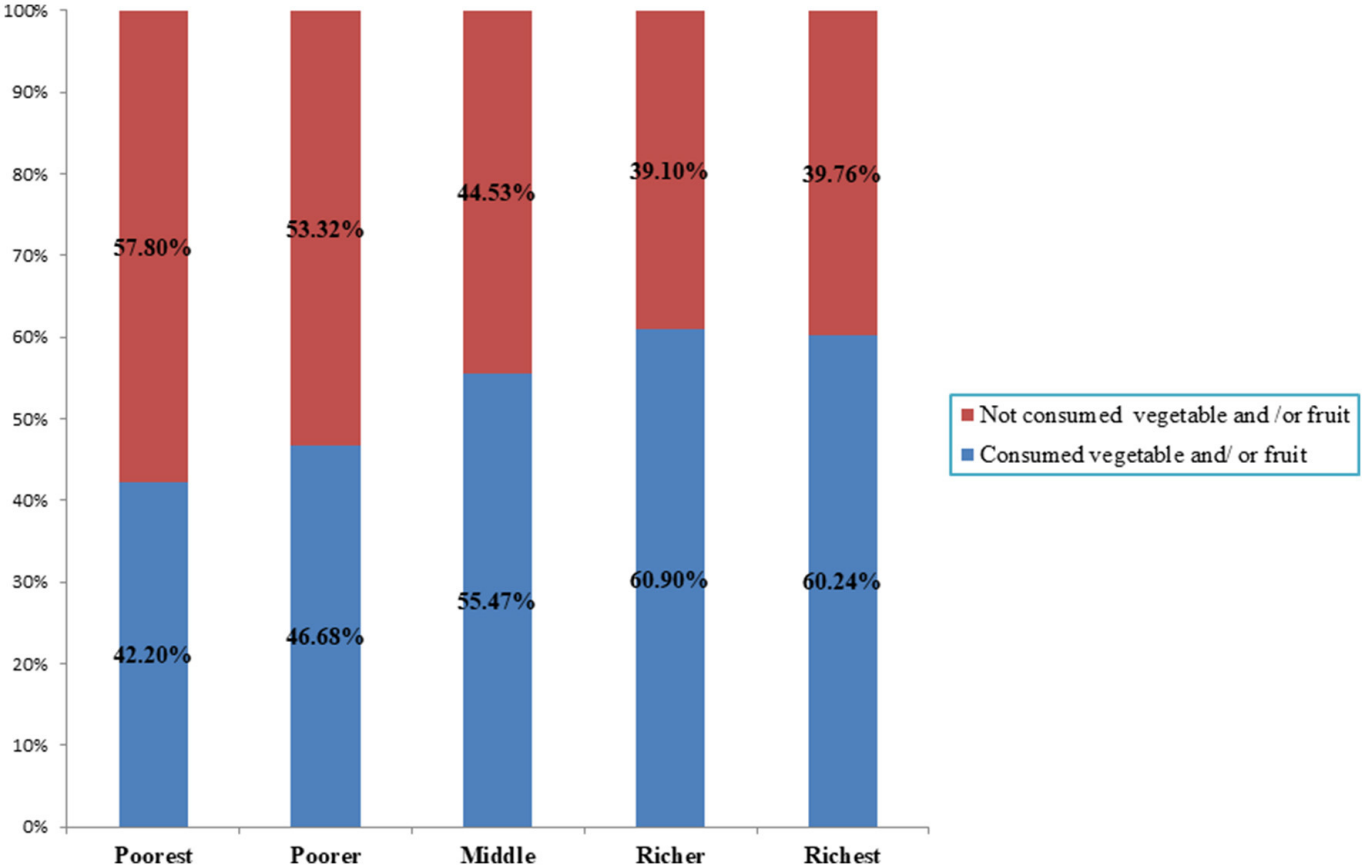
Zero vegetable and /or fruit consumption among children aged 6–23 month in east African countries using recent demographics and health survey across household wealth index.

**Figure 3:**
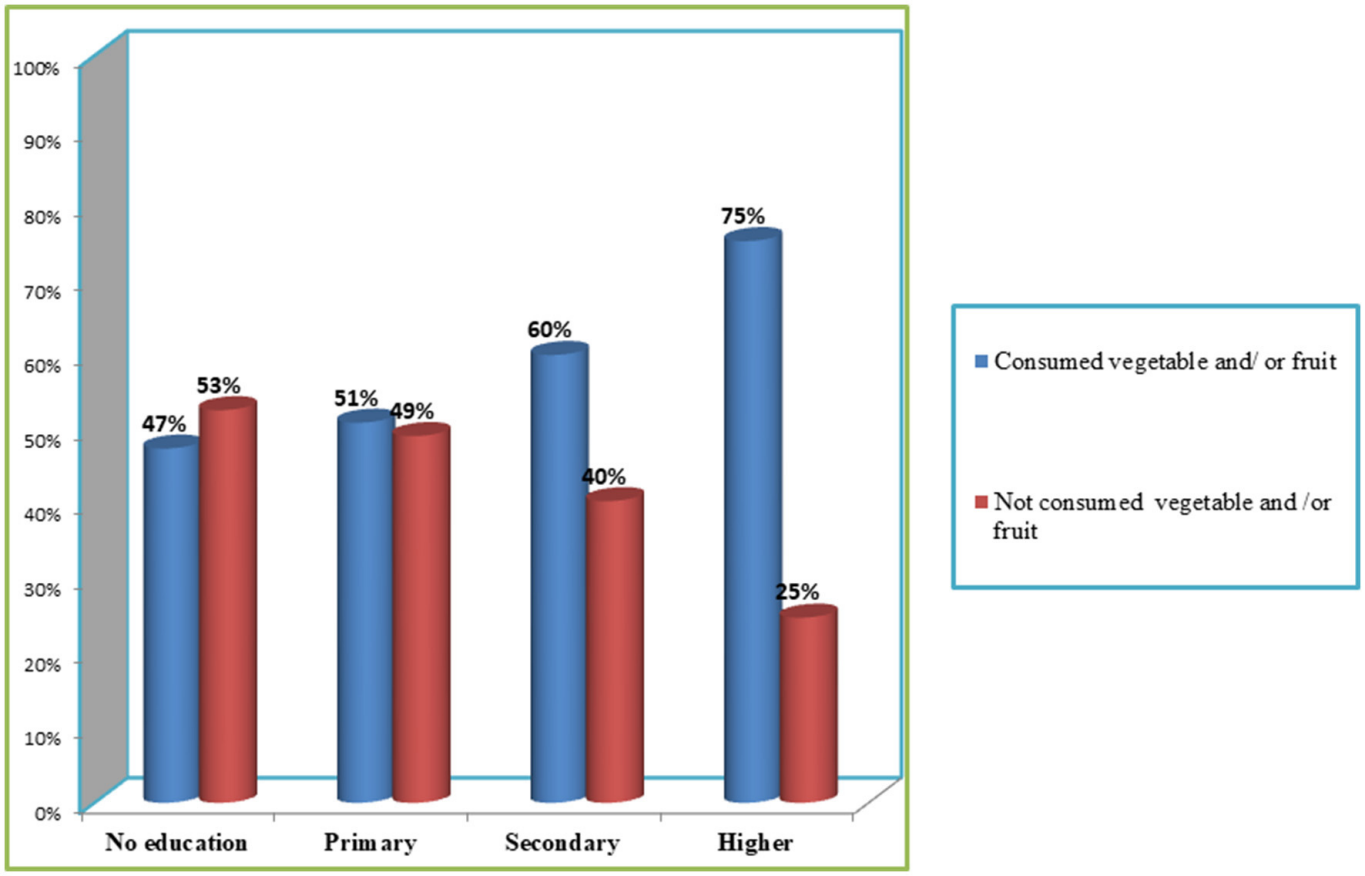
Zero vegetable and /or fruit consumption among children aged 6–23 month in east African countries using recent demographics and health survey across maternal level of educational status.

Figures 2, 3 illustrate the percentage of children aged 6–23 months in East African countries who do not consume any vegetables or fruits based on the household wealth index and maternal educational status, respectively. According to the data, 57.8% of the poorest children have ZVF consumption, whereas 60.2% of the richest children consume vegetables and fruits (Figure 2). Regarding maternal educational status, 53% of children whose mothers are not educated have ZVF consumption, whereas 75% of children whose mothers have higher education levels consume vegetables and fruits (Figure 3).

### Effects of household- and community-level factors on ZVF consumption

In Model-5, maternal educational status, birth interval, multiple births, number of household members, sex of the household head, household wealth index, community-level maternal literacy, community-level wealth index, and countries had a statistical association with ZVF consumption. Children whose mothers have primary, secondary, and higher education, respectively, were 20% [odds ratio (OR): 0.8, 95% confidence interval (CI) (0.75, 0.85)], 28% [OR: 0.72, 95% CI (0.7, 0.8)], and 40% [OR: 0.6, 95% CI (0.46, 0.65)] less likely to have ZVF consumption than those whose mothers have no education. Children living with mothers who were in union were 20% less likely to have ZVF consumption than those whose mothers were in un-union [OR: 0.8, 95% CI (0.86, 0.93)]. ZVF consumption was 1.3 times more likely to be observed in children who live with more than seven household members than in children who live with < 5 household members [OR: 1.3, 95% CI (1.2, 1.35)]. The ZVF consumption was 30% and 10% less likely among children whose birth intervals equal to and > 2 years than among children whose birth intervals were < 2 years [OR: 0.7, 95% CI (0.6, 0.8)] and [OR: 0.9, 95% CI (0.8, 0.98)], respectively. ZVF consumption was 1.4 times more likely to be observed in children whose mothers had multiple births than those whose mothers had single births [OR: 1.4, 95% CI (1.2, 1.7)]. ZVF consumption was 30% and 25% less likely among children from average-income and rich households than from those from poor households [OR: 0.7 95% CI (0.69, 0.8)] and [OR: 0.75, 95% CI (0.7, 0.8)], respectively. ZVF consumption was two times more likely among children from low-wealth communities than those from high-wealth communities [OR: 2, 95% CI (1.4, 2.8)]. ZVF consumption was 72% less likely among children with a high proportion of educated mothers in the community as compared to their counterparts [OR: 0.18, 95% CI (0.12, 0.3)]. ZVF consumption was 22, 11.3, 4.19, 1.8, 8.2, and 5.2 times more likely among children from Ethiopia, Kenya, Comoros, Rwanda, Tanzania, and Uganda than among those from Burundi [OR: 22, 95% CI (19.7, 25.3)], [OR: 11.3, 95% CI (10.12, 12.8.)], [OR: 4.19, 95% CI (3.6, 4.78)], [OR: 1.8, 95% CI (1.6, 2)], [OR: 8.2, 95% CI (7.4, 8.9)], and [OR: 5.2, 95% CI (4.7, 5.6)], respectively (Table 3).

**Table 3:** Bivariable and multilevel mixed effect logistic regression analysis of household- and community-level factors of zero vegetable or fruit consumption among children aged 6–23 months in East Africa based on DHS data from 2015–2023.

**Variable**	**Category**	**Bivariable 95% COR**	**Model-0**	**Model-1**	**Model-2**	**Model-3**	**Model-4**	**Model-5**
			**ICC** = **80%**	**95% AOR**	**95% AOR**	**95% AOR**	**95% AOR**	**95% AOR**
Maternal age	15–19	Reference						
	20–24	0.7 (0.08, 5.6)		0.7 (0.09, 05.7)				0.6 (0.05–6.2)
	25–29	0.2 (0.03, 1.7)		0.3 (0.0, 1.5)				0.22 (0.022, 2.3)
	30–34	0.22 (0.03, 1.7)		0.04 (0.03, 1.6)				0.24 (0.024, 2.5)
	35–39	0.18 (0.02, 1.5)		0.23 (0.02, 1.3)				0.22 (0.02, 2.3)
	40–45	0.14 (0.02, 1.1)		0.112 (0.1, 0.9)				0.16 (0.016, 1.5)
	45–49	0.12 (0.02, 1.02)		0.12 (0.01, 0.8)				0.14 (0.014, 1.5)
Maternal educational status	No education	Reference						
	Primary education	0.8 (0.75, 0.83)		0.76 (0.7.0.8)				0.8 (0.75, 0.85)^*^
	Secondary education	0.77 (0.7, 0.84)		0.7 (0.6, 0.8)				0.72 (0.7, 0.8)^*^
	Higher education	0.55 (0.46, 0.64)		0.5 (0.47, 0.7)				0.6 (0.46, 0.65)^*^
Marital status	Un-union	Reference						
	Union	0.69 (0.63, 0.75)		0.68 (0.6, 0.7)				0.8 (0.76, 0.93)^*^
Number of household members	1–5	Reference						
	6–7	0.8 (0.7, 0.88)		0.94 (0.88, 1)				0.99 (0.9, 1.06)
	>7	1.1 (1.06, 1.2)		1.4 (1.3, 1.5)				1.3 (1.2, 1.35)^*^
Number of children	< =4	Reference						
	>4	1.3 (1.1, 1.6)		1.0 (0.79, 1.28)				0.5 (0.43, 1.07)
Birth interval	< 2 years	Reference						
	=2 years	0.61 (0.54, 0.68)		0.6 (0.55, 0.69)				0.7 (0.6, 0.8)^*^
	>2 years	0.84 (0.8, 0.88)		0.9 (0.83, 0.92)				0.9 (0.8, 0.98)^*^
Birth type	Single	Reference						
	Multiple	1.34 (1.1, 1.5)		1.37 (1.15, 1.6)				1.4 (1.2, 1.7)^*^
Sex of the child	Boy	Reference						
	Girl	0.94 (0.9, 0.98)		0.94 (0.9, 0.99)				0.9 (0.8, 1.9)
Age of the child	6–11 months	Reference						
	12–24 months	0.9 (0.89, 0.97)		1.1 (1, 1.15)				0.99 (0.9, 1.04)
Sex of the household head	Man	Reference						
	Woman	1.2 (1.15, 1.29)			1.1 (0.8, 1.2)		1.1 (1.03, 1.2)	1.1 (1.03, 1.18)^*^
Age of the household head	15–21	Reference						
	22–34	0.3 (0.24, 0.67)			0.3 (0.1, 0.7)		0.4 (0.2, 0.8)	0.5 (0.2, 1.104)
	35–64	0.26 (0.12, 0.58)			0.3 (0.1, 0.6)		0.3 (0.2, 0.7)	0.5 (0.2, 1.12)
	>64	0.5 (0.23, 1.3)			0.5 (0.2, 1.2)		0.4 (0.2, 1.8)	0.65 (0.29, 1.5)
Household wealth index	Poor	Reference						
	Average	0.68 (0.64.0.72)			0.7 (0.6, 0.8)		0.7 (0.69, 0.8)	0.76 (0.7, 0.8)^*^
	Rich	0.58 (0.54, 0.6)			0.6 (0.5, 0.6)		0.75 (0.7, 0.8)	0.8 (0.76, 0.86)^*^
Community-level wealth index	Low	Reference						
	High	2.7 (1.9, 3.8)				2 (1.2, 2.8)	1.7 (1.2, 2.4)	2 (1.4, 2.8)^*^
Community-level maternal literacy	Low	Reference						
	High	0.33 (0.23, 0.48)				0.2 (0.1, 0.3)	0.2 (0.1, 0.3)	0.18 (0.12, 0.3)^*^
Country	Burundi	Reference						
	Ethiopia	25.4 (22.3, 28.7)				25 (21, 27)	24.5 (21, 26)	22 (19.7, 25.3)^*^
	Kenya	10.9 (9.7, 12.35)				11 (10, 12)	10.8 (9.6, 12)	11.3 (10, 12.8)^*^
	Comoros	4.8 (4.2, 5.46)				4.8 (4.2, 5.4)	4.4 (3.8, 5.4)	4.19 (3.6, 4.78)^*^
	Rwanda	1.5 (1.4, 1.7)				1.7 (1.4, 1.6)	1.5 (1.4, 1.6)	1.8 (1.6, 2)^*^
	Tanzania	7.9 (7.2, 8.6)				7.9 (7.2.8.6)	7.6 (6.9, 8.3)	8.2 (7.4, 8.9)^*^
	Uganda	5.2 (4.8, 5.5)				5.2 (4.8, 5.5)	4.9 (4.6, 5.2)	5.2 (4.7, 5.6)^*^

^*^Significant at p < 0.05, AOR, adjusted odds ratio; COR, Crud odds ratio; ICC, intra-class correlation; Model-0, empty model or null model conducted without an independent variable (univariate analysis; the result showed ICC = 80%); Model-1, analysis of only individual-level variables; Model-2, analysis of only household-level variables; Model-3, analysis of only community-level variables; Model-4, analysis of only household- and community-level variables; and Model-5, analysis of all individual-, household-, and community-level variables based on cutoff points.

### Random effects (measurement of variation)

ZVF consumption among children aged 6–23 months in East Africa varies significantly across each cluster (i.e., household, community, or country). Intra-cluster correction coefficient (ICC) indicated that 80% of the variation in ZVF consumption among children aged 6–23 months was attributed to community-level factors. Proportional change of variability in the final model indicated that 4.3% of the variation in ZVF consumption among children aged 6–23 months was attributed to communities or countries. The MOR confirmed that the variation in ZVF consumption was affected by community-level factors. In the initial model (Model-0), the MOR for ZVF consumption was 4.382, indicating significant variation between communities (4.382 times higher than the reference or MOR = 1). However, when all factors were added to the model (Model-5), the unexplained community variation decreased with an MOR of 4.126. This finding suggests that, even after considering all factors, the effects of clustering remain statistically significant in the full models (Table 4).

**Table 4:** Model building and model selection.

**Model building and selection**	**Model-0**	**Model-1**	**Model-2**	**Model-3**	**Model-4**	**Model-5**
Variance	11.6	11.58	11.28	11.36	11.27	11.24
ICC	80%	79%	77.48%	77.8%	77.45%	77.1%
PCV	Reference	0.2%	2.7%	2%	2.8%	3.1%
MOR	4.382	4.33	4.2097	4.2096	4.185	4.126
AIC	82893.550	81848.050	82333.510	76863.190	76678.140	76255.550
BIC	82912.900	82012.540	82410.910	76959.940	76832.950	76555.500

AIC, Akaike information criterion; BIC, Bayesian information criterion; ICC, intra-class correlation; MOR, median odds ratio; PCV, proportional change of variability. Model-0, empty model or null model conducted without an independent variable (univariate analysis; the result showed ICC = 80%); Model-1, analysis of only individual-level variables; Model-2, analysis of only household-level variables; Model-3, analysis of only community-level variables; Model-4, analysis of only household- and community-level variables; and Model-5, analysis of all individual-, household-, and community-level variables based on cutoff points.

## Discussion

This study investigated the household- and community-level factors determining ZVF consumption among children aged 6–23 months in East Africa based on the new indicators developed by WHO and UNICEF for assessing feeding practices for infants and young children (1).

In East Africa, the overall prevalence of ZVF consumption among children aged 6–23 months was 52.3%. Ethiopia had the highest percentage (85.9%) and Rwanda had the lowest (30.3%). This disparity can be attributed to differences in child-feeding practices followed in these countries, such as complementing feeding programs (27).

The overall prevalence of ZVF consumption in this study was higher than that found in a multi-country study conducted on children aged 6–23 months (45%) (12) and that found in a study conducted in SSA countries (47%). The low consumption of vegetables or fruits in all East African countries may be due to low affordability and scarcity. However, the prevalence of ZVF consumption in West and Central Africa (56.1%) (12) was higher in a previous study than in this study. This increase in ZVF consumption in these regions may be because of environmental changes and socioeconomic variation as well as the inadequacy of child health services, such as complementing feeding programs (27).

Maternal educational status, short birth interval, multiple births, number of household members, sex of the household head, household wealth index, community-level maternal educational status, community-level wealth index, and countries had a statistical association with ZVF consumption. These findings are supported by the findings of previous studies (4, 8, 12, 13). Children whose mothers have higher educational status were more likely to consume fruits and vegetables, which is supported by previous findings, for example, a study conducted in the Netherlands found that children of mothers with higher educational levels consumed more fruits and vegetables per day than children of mothers with lower educational levels (28). This effect of educational level could be because mothers with higher educational levels are likely to be more aware of the importance of healthy eating habits and are better equipped to provide their children with healthy food options. Conversely, uneducated mothers may lack an understanding of child-feeding practices.

Children from households with more numbers of members were less likely to consume fruits and vegetables compared to those with fewer members. This result is consistent with other study findings. For instance, a study conducted in the United Kingdom found that the number of household members was negatively associated with total fruit and vegetable consumption among children aged 6–23 months (29). This trend could be because, with a higher number of members, the household may face difficulty in accessing healthy food options or each member may have different dietary preferences. It is important to note that the higher number of household members potentially leading to a shortage of food items is just one of the many factors that can influence a child's eating habits. Other factors such as household income, access to healthy food options, and cultural practices may also play a role, as indicated by the strong association between the number of household members and ZVF consumption for children aged 6–23 months in this study.

This study found that the short birth interval and types of birth of children are associated with ZVF consumption, which is also supported by a study conducted in Ethiopia (8).

Children born to mothers with multiple births were more likely to have ZVF consumption compared to those born to mothers with single births. The findings of previous studies in Denver metro (30) and Brazil (31) confirm the same trend.

Child with households with a female head were less likely to consume fruits and vegetables than those with a male head. This finding can be attributed to women having less decision-making power and mobility in households with a male head, restricting them from visiting marketplaces and purchasing food (32). This is a common issue in many households where women do not have equal opportunities to make decisions and have limited mobility, leading to a lack of access to healthy food options, which in turn negatively impacts the health of children. It is important to address this issue by empowering women and providing them with the resources that they need to make informed decisions about their family's health and wellbeing.

According to this study, children in households with higher incomes had a higher consumption of vegetables and fruits than those in lower-income households. This finding is supported by studies conducted in Ethiopia (8), Ghana (18), and SSA countries (13). The possible reason for this trend may be that children from lower-income households may have limited access to fruits and vegetables because of economic constraints, leading them to possibly sell fruits and vegetables to buy cheaper foods (33). This study suggests that children living in communities with lower educational levels are more likely to have ZVF consumption, a finding supported by several other studies (13, 15, 16). The possible reason for this trend may be that communities with lower education levels may lack necessary dietary knowledge and feeding practices, leading to less likelihood of consuming diversified foods in the community.

Children from Ethiopia, Kenya, Comoros, Rwanda, Tanzania, and Uganda were more likely to have ZVF consumption compared to children from Burundi. The overall ZVF consumption among children aged 6–23 months varies significantly across households, communities, or countries. The concerned organizations in these countries should collaborate to improve fruit and vegetable consumption through increased education.

### Practical and policy implications

The study highlights the need for targeted education programs aimed at improving maternal literacy within communities. These educational programs can include sharing information on the importance of consuming fruits and vegetables for children's growth and development. The findings of this study can inform the development of national policies and strategies to address the issue of ZVF consumption among children. Overall, the findings of this study emphasize the importance of multilevel interventions, including household-, community-, and policy-level approaches, to promote vegetable and fruit consumption among children in East Africa.

## Strength and limitation

A major strength of this study is the use of nationally representative data, which allows it to be generalizable to children in all East African countries. However, the DHS surveys are conducted every 5 years, and the data may not reflect real-time changes or trends in child health and nutritional issues. In addition, the data collection through DHS surveys relies on self-reported information, which can be subject to recall bias or social desirability bias.

## Directions for future research

Future researchers should consider longitudinal studies to observe changes in vegetable and fruit consumption among children over time. To complement the quantitative analysis, researchers should incorporate qualitative research methods to gain a deeper understanding of the cultural, social, and economic factors that affect ZVF consumption in East Africa.

## Conclusion

The overall prevalence of zero vegetable or fruit (ZVF) consumption among children aged 6–23 months in East Africa was high, with Ethiopia having the highest prevalence. The factors affecting this high ZVF consumption trend are individual-level factors such as maternal educational level, short birth interval, and multiple births; household-level factors such as the number of household members, sex of the household head, and household wealth index; and community-level factors such as maternal literacy, community-level wealth index, and countries. This study highlights the issue of high ZVF consumption, and overlooking this nutritional gap in children is a serious oversight. Therefore, mothers, communities, and governments of each country should collaborate to improve fruit and vegetable consumption by increasing maternal educational level and promoting equitable economic opportunities for low-income households. Emphasizing child nutrition, including fruit and vegetable consumption, should be a priority at all health education levels and across health sectors. In particular, nutrition and public health organizations should support low-income communities to achieve diet diversity for infants and young children.

## Data availability statement

Publicly available datasets were analyzed in this study. This data can be found at: https://dhsprogram.com.

## Ethics statement

Ethical approval was not required for the study involving humans in accordance with the local legislation and institutional requirements. Written informed consent to participate in this study was not required from the participants or the participants' legal guardians/next of kin in accordance with the national legislation and the institutional requirements.

## Author contributions

AE: Conceptualization, Data curation, Formal analysis, Funding acquisition, Investigation, Methodology, Project administration, Resources, Software, Supervision, Validation, Visualization, Writing – original draft, Writing – review & editing. AG: Writing – original draft, Writing – review & editing. AMu: Writing – original draft, Writing – review & editing. AMo: Writing – original draft, Writing – review & editing. AA: Writing – original draft, Writing – review & editing. DM: Writing – original draft, Writing – review & editing. EA: Writing – original draft, Writing – review & editing. FDB: Writing – original draft, Writing – review & editing. FB: Writing – original draft, Writing – review & editing. LA: Writing – original draft, Writing – review & editing.
